# Unraveling the Potential Role of *Tecomella undulata* in Experimental NASH

**DOI:** 10.3390/ijms24043244

**Published:** 2023-02-07

**Authors:** Akshatha N. Srinivas, Diwakar Suresh, Deepak Suvarna, Pankaj Pathak, Suresh Giri, Suman, Suchitha Satish, Saravana Babu Chidambaram, Divya P. Kumar

**Affiliations:** 1 Department of Biochemistry, Center of Excellence in Molecular Biology and Regenerative Medicine (CEMR), JSS Medical College, JSS Academy of Higher Education and Research, Mysuru 570004, India; 2 Department of Gastroenterology, JSS Medical College and Hospital, JSS Academy of Higher Education and Research, Mysuru 570004, India; 3 JSS Ayurveda Medical College and Hospital, Mysuru 570028, India; 4 Zydus Research Centre, Cadila Healthcare Limited, Ahmedabad 382213, India; 5 Government Ayurveda Medical College, Mysuru 570001, India; 6 Department of Pathology, JSS Medical College and Hospital, JSS Academy of Higher Education and Research, Mysuru 570004, India; 7 Department of Pharmacology, JSS College of Pharmacy, JSS Academy of Higher Education and Research, Mysuru 570004, India

**Keywords:** insulin resistance, hepatic inflammation, oxidative stress, ER stress, herbal medicine

## Abstract

The pathophysiology of nonalcoholic steatohepatitis (NASH) is complex, owing to its diverse pathological drivers and, until recently, there were no approved drugs for this disease. *Tecomella* is a popular herbal medicine used to treat hepatosplenomegaly, hepatitis, and obesity. However, the potential role of *Tecomella undulata* in NASH has not yet been scientifically investigated. The administration of *Tecomella undulata* via oral gavage lowered body weight, insulin resistance, alanine transaminase (ALT), aspartate transaminase (AST), triglycerides, and total cholesterol in western diet sugar water (WDSW) fed mice but had no effect on chow diet normal water (CDNW) fed mice. *Tecomella undulata* improved steatosis, lobular inflammation, and hepatocyte ballooning and resolved NASH in WDSW mice. Furthermore, *Tecomella undulata* also alleviated the WDSW-induced Endoplasmic Reticulum stress and oxidative stress, enhanced antioxidant status, and thus reduced inflammation in the treated mice. Of note, these effects were comparable to saroglitazar, the approved drug used to treat human NASH and the positive control used in the study. Thus, our findings indicate the potential of *Tecomella undulata* to ameliorate WDSW-induced steatohepatitis, and these preclinical data provide a strong rationale for assessing *Tecomella undulata* for the treatment of NASH.

## 1. Introduction

Nonalcoholic fatty liver disease (NAFLD) is a multisystemic disease that encompasses a spectrum of conditions, including benign fatty liver, nonalcoholic steatohepatitis (NASH), fibrosis, cirrhosis, and hepatocellular carcinoma [1]. NAFLD, a significant health burden caused by the rise in obesity and sedentary lifestyle, is estimated to have a 25% global prevalence and is expected to quadruple by 2030 if management is inadequate [2]. The fatty liver resulting from overnutrition and insulin resistance promotes proinflammatory settings for NASH [3]. The liver is overworked as a result of increased hepatic intake, decreased lipolysis, and de novo lipogenesis, which results in lipotoxicity, an inflammatory response, cell death, and fibrogenic activation [4]. Thus, despite a plethora of studies, the pathobiology of NASH is poorly understood due to its diverse disease drivers, leading to heterogeneous therapeutic responses. There are still unmet medical needs and challenges and, until recently, there were no approved medications for NAFLD. However, in 2020, saroglitazar became the first medication for NASH to receive approval and be prescribed to patients in India [5].

The current paradigm of multiple hits involving a number of factors acting in parallel provides a better delineation of NASH development and progression [6]. Oxidative stress is a major contributor among the multiple hits of NASH progression. The imbalance between the production and scavenging of reactive oxygen species (ROS) due to hepatic lipid overload leads to metabolic dysfunction, followed by an inflammatory response [7]. The accumulation of unfolded proteins in the endoplasmic reticulum (ER) lumen as a result of lipotoxicity causes ER stress, which is characterized by elevated levels of ER stress markers and mediates the inflammatory cascades in the progression of NASH [8,9]. In contrast, antioxidant levels are significantly downregulated in NASH patients, reiterating the root cause of disease progression [10]. Furthermore, the disease’s progression is determined by the cellular stress-mediated inflammatory tone caused by immune cell crosstalk [11] As a result, the discovery of drugs with pleiotropic effects is an effective therapeutic strategy for combating the disease.

In recent years, plant-based medicines have gained attention, along with plant-based dietary patterns. Whole medicinal plants, which are unpurified extracts, have been traditionally used in many parts of the world as an effective therapy for NAFLD management [12]. Holistic approaches to herbal medicine have benefits, since they allow for the identification of pure natural ingredients in these medications. *Tecomella undulata* (Sm.) Seem. (Rohitaka) (The plant name has been verified with World Flora Online (http://www.worldfloraonline.org (accessed on accessed on 20 July 2021)) and MPNS (https://mpns.science.kew.org (accessed on accessed on 20 July 2021))) is one such traditional medicine belonging to the Bignoniaceae family, which is widely used in Ayurveda for its pharmacological effects including hepatoprotection, immunomodulation, anti-inflammatory, and antimicrobial activity [13,14,15]. *Tecomella undulata*, a native shrub of northeastern and western India, has also shown promise as an anti-obesity formulation [16]. The stem bark powder of *Tecomella undulata* is widely used in treating ascites with hepatosplenomegaly and also in hepatitis, as it is known to work efficiently as a blood purifier [17]. Of note, the stem bark powder of *Tecomella undulata* is a key ingredient in many herbal remedies for the treatment of inflammatory liver illnesses, including Livo-plus, Liv-52, Herboliv, Livfit, Amylcure, Himoliv, Livosan, SLIM-U capsules and Exol [18]. Experimental studies have shown the hepatoprotective potential of *Tecomella undulata* against paracetamol-, carbon tetrachloride-, and thioacetamide-induced hepatotoxicity [19]. Thus, the medicinal value with effective pharmacological effects of *Tecomella undulata* has drawn a lot of interest in the liver field. However, the potential role of *Tecomella undulata* and its molecular mechanisms in NASH has not been investigated. 

The overall goal of this study was to investigate the pharmacological effects of *Tecomella undulata* and offer solid justification for its use in the clinical treatment of NASH. We here report the effects of *Tecomella undulata* on the pathogenesis of NASH using a mouse model (detailed in Section 4) that mimics the key elements of human NASH in terms of histological progression and molecular signature [20,21,22]. The specific objectives of the study were to demonstrate the efficacy of *Tecomella undulata* on (i) systemic obesity and insulin resistance, (ii) inflammation, and (iii) key pathogenic signaling pathways in NASH. Taken together, these provide novel data demonstrating scientific evidence for the traditional claim of *Tecomella undulata*’s hepatoprotective action in the context of NASH. 

## 2. Results

### 2.1. Quality Evaluation and Dose Selection of Tecomella undulata for Preclinical Studies

As per the guidelines of ‘Quality control methods for medicinal plant materials’ by the World Health Organization (WHO) (https://www.who.int/publications/m/item/quality-control-methods-for-medicinal-plant-materials (accessed on 1 September 2021)) and the Ayurvedic Pharmacopoeia of India (API), Government of India [23], the raw/crude stem bark powder of *Tecomella undulata* was subjected to pharmacopoeia tests. Various standardization components that were analyzed include macroscopic and microscopic characteristics, organoleptic characteristics, physiochemical properties, presence of residues (heavy metals, pesticides, toxins), and microbial load (Supplementary Table S1). After determining that the values were within the limits or matched with the pharmacopoeial standards of WHO and API, *Tecomella undulata* powder was used for the mice study. Furthermore, in vitro cell viability assays were also performed prior to preclinical studies (Supplementary Figure S2). 

The choice of *Tecomella undulata* dosage was determined based on the human therapeutic dose and body surface area conversion factor, as described in Section 4. A dose of 80 mg/kg body weight of mice was chosen for the in vivo experiments. 

### 2.2. Tecomella undulata Treatment Ameliorates Western Diet Sugar Water-induced Obesity and Insulin Resistance

**Figure 1:**
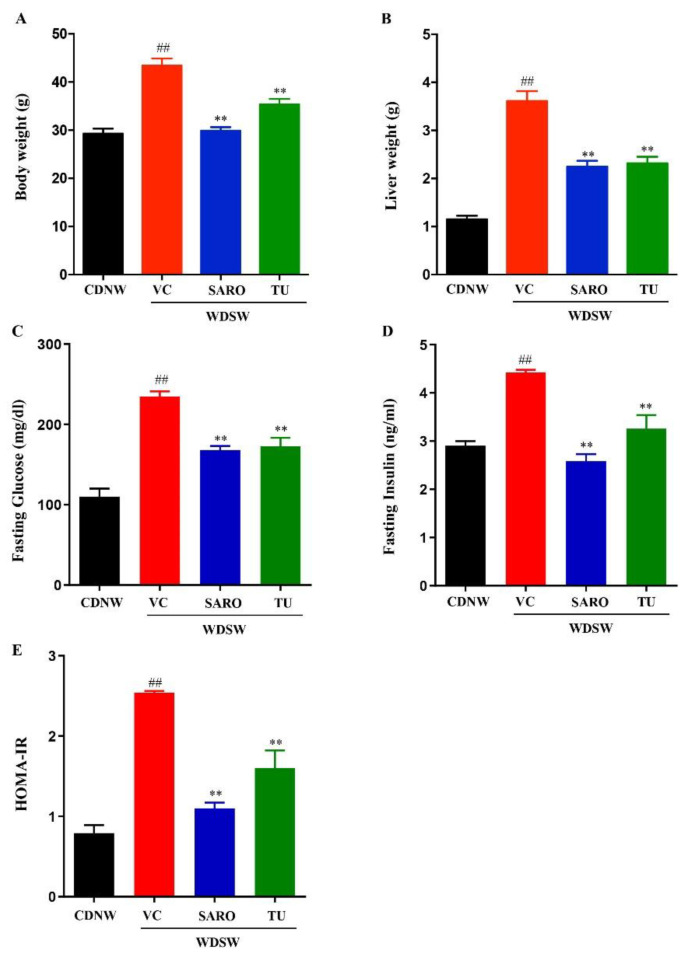
***Tecomella undulata*** (**TU**) **reduced body weight and improved insulin resistance in high fat diet-induced obese mice.** Mice were treated with CDNW or WDSW for 12 weeks and, following that, WDSW mice were divided into 3 groups and treated with (i) vehicle control (VC) (ii) saroglitazar (SARO) (iii) *Tecomella undulata* (TU) via oral gavage for additional 12 weeks. At the completion of the treatment, body weight (**A**), liver weight (**B**), fasting glucose (**C**), and fasting insulin (**D**) were measured. HOMA-IR (**E**) was calculated using the formula: (fasting insulin (milliunits/liter) × fasting glucose (mmol/liter))/22.5. Data are expressed as mean with SEM for 8–10 mice per group. ## *p* < 0.001 compared to CDNW; ** *p* < 0.001 compared to WDSW, vehicle control.

The C57Bl/6 mice were divided into two groups and fed with chow diet normal water (CDNW) and western diet sugar water (WDSW), respectively. The mice were further divided into treatment groups for vehicle control, saroglitazar (4 mg/kg body weight), and *Tecomella undulata* (80 mg/kg body weight). Mice fed with WDSW became obese, with increased body weight and liver weight compared to mice fed with CDNW. Additionally, WDSW mice also developed insulin resistance. The mice treated with *Tecomella undulata* for 12 weeks showed a significant reduction in body weight and liver weight compared to the vehicle control (Figure 1A,B and Supplementary Figure S3A,B). In addition, calorie intake was not affected by *Tecomella undulata* treatment, which indicates that the decrease in body weight by treatment was not due to changes in food consumption. *Tecomella undulata* also significantly reduced fasting glucose and fasting insulin levels in WDSW fed mice (Figure 1C,D). Insulin resistance, a key driver of NASH, was also reduced by *Tecomella undulata* treatment, as measured by HOMA-IR (homeostatic model assessment for insulin resistance) (Figure 1E). 

**Figure 2:**
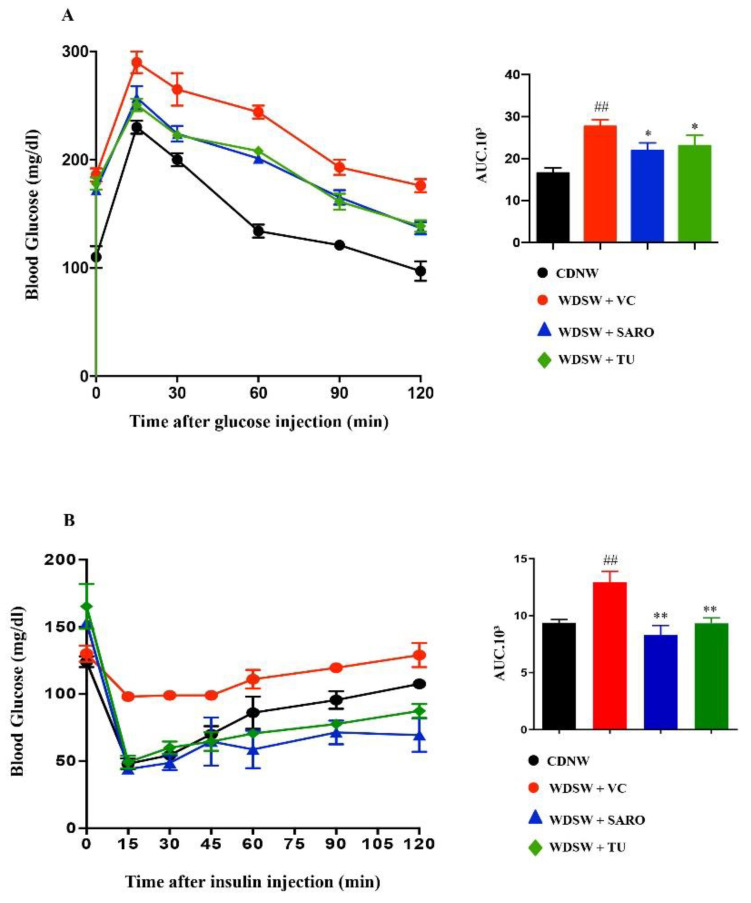
***Tecomella undulata*** (**TU**) **improves glucose tolerance and insulin sensitivity.** CDNW and WDSW mice treated with vehicle control (VC), saroglitazar (SARO) or *Tecomella undulata* (TU) for 12 weeks. Mice were fasted overnight and blood glucose concentration (mg/dl) was measured after intraperitoneal injection of 1 g/kg glucose (**A**). Mice fasted for 4–5 h were administered 0.75 units/kg insulin and blood glucose concentration (mg/dL) measured (**B**). The bar graphs depict the area under the curve (AUC) with or without treatment. Data are expressed as mean with SEM for 8–10 mice per group. ## *p* < 0.001 compared to CDNW; ** *p* < 0.001 or * *p* < 0.05 compared to WDSW, vehicle control.

Additionally, administration of *Tecomella undulata* improved glucose tolerance (assessed by a glucose tolerance test) and insulin sensitivity (assessed by an insulin tolerance test) in hyperglycemic WDSW mice compared with the vehicle alone (Figure 2A,B), but had no effect in CDNW mice (Supplementary Figures S4 and S5). Overall, the degree of amelioration was equivalent to saroglitazar, the positive control group used in the study. 

### 2.3. Tecomella undulata Prevented Liver Injury and Hyperlipidemia

**Figure 3:**
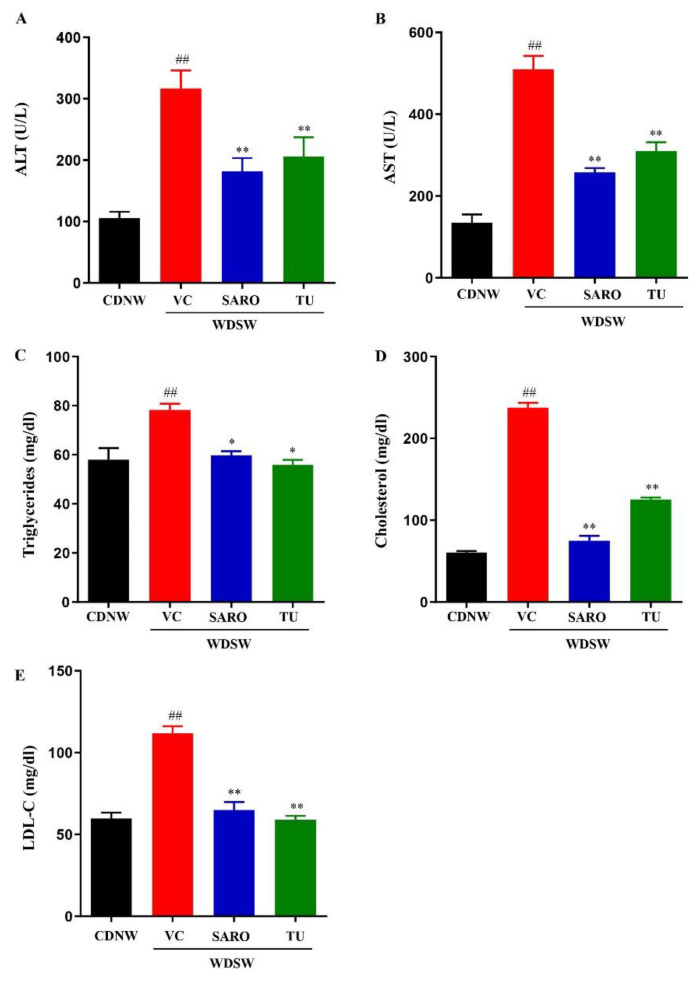
***Tecomella undulata*** (**TU**) **treatment reduced liver injury and hyperlipidemia.** CDNW or WDSW mice, after 12 weeks, were administered vehicle control (VC), saroglitazar (SARO) or *Tecomella undulata* (TU) for another 12 weeks. At the end of 24 weeks following dietary intervention and treatment, mice were fasted overnight and blood was collected. (**A**) serum ALT, (**B**) serum ALT, (**C**) serum triglycerides, (**D**) serum cholesterol (**E**) serum LDL-C. Data are expressed as the mean with SEM for 8–10 mice per group. ## *p* < 0.001 compared to CDNW; ** *p* < 0.001 or * *p* < 0.05 compared to WDSW, vehicle control. AST, aspartate aminotransferase; ALT, alanine aminotransferase; LDL-C, low-density lipoprotein-cholesterol.

As expected, the WDSW diet drastically elevated liver enzymes and the mice developed hyperlipidemia, an indicator of liver injury. However, *Tecomella undulata* treatment in these mice led to a reduction in the serum levels of ALT and AST (Figure 3A,B). In addition, *Tecomella undulata* administration also improved circulating triglycerides, total cholesterol, and LDL-cholesterol in WDSW mice compared to the vehicle control group (Figure 3C–E). Consistently, *Tecomella undulata* had no effect on the liver enzymes and lipid profile of CDNW mice, as is the case with saroglitazar (Supplementary Figure S6). 

Collectively, these data demonstrate that *Tecomella undulata* significantly ameliorates western diet sugar water-induced obesity, insulin resistance, liver injury, and dyslipidemia. 

### 2.4. Tecomella undulata Improves Hepatic Steatosis and Steatohepatitis 

**Figure 4:**
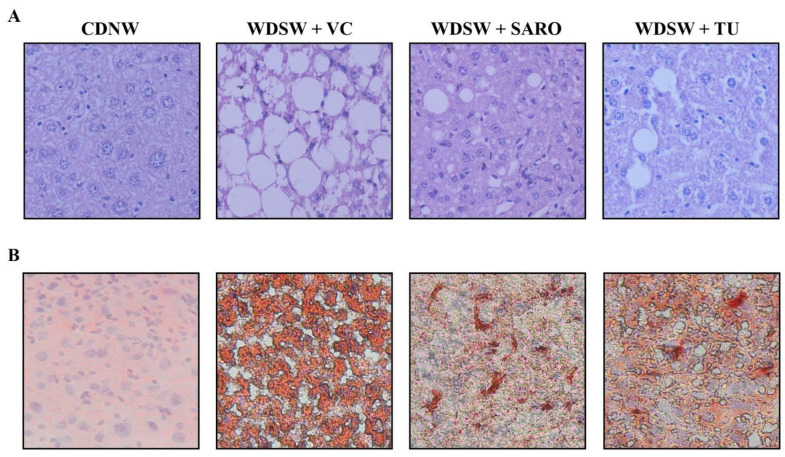
**Histological features of mice treated with *Tecomella undulata*** (**TU**)**.** Representative microscopic views of liver sections from CDNW or WDSW mice treated with vehicle control (VC), saroglitazar (SARO) or *Tecomella undulata* (TU). Intracellular lipid accumulation was determined by staining with (**A**) hematoxylin and eosin (H&E), and (**B**) Oil Red O.

**Figure 5:**
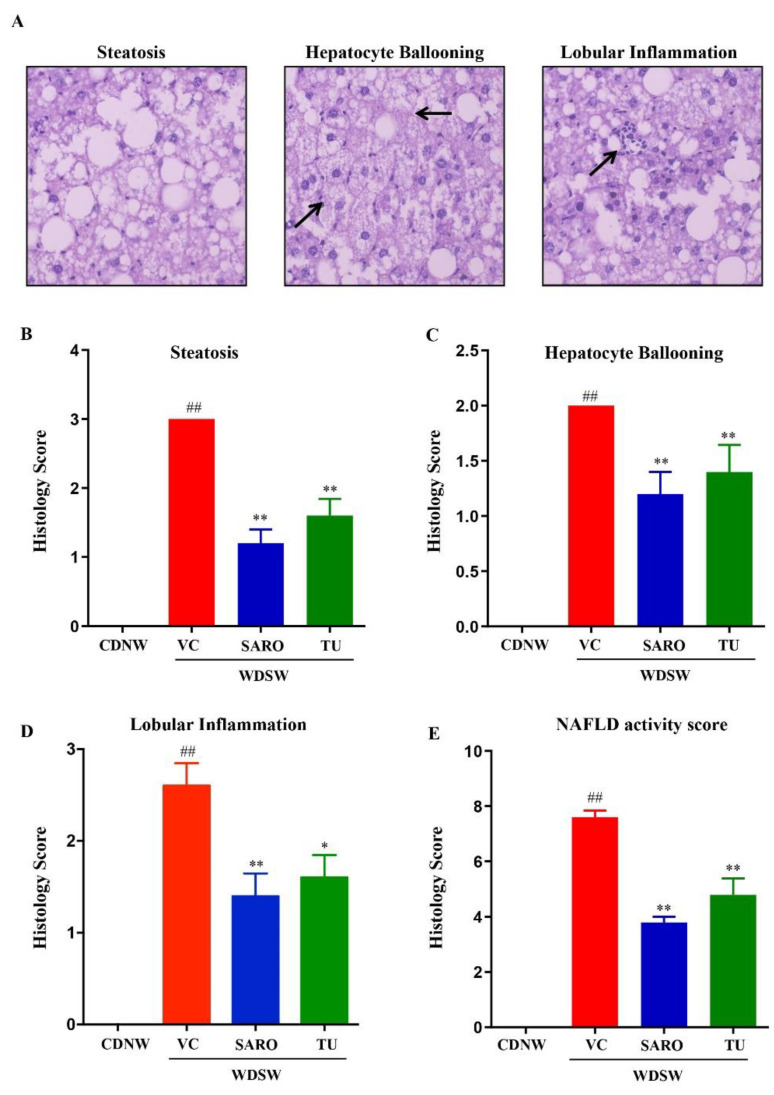
***Tecomella undulata*** (**TU**) **treatment ameliorates fatty liver and steatohepatitis.** CDNW or WDSW mice treated with vehicle control (VC), saroglitazar (SARO) or *Tecomella undulata* (TU) for 12 weeks. (**A**) hematoxylin and eosin (H&E) stained liver sections depicting steatosis, hepatocyte ballooning, and lobular inflammation. Histology score for (**B**) steatosis, (**C**) hepatocyte ballooning, (**D**) lobular inflammation, (**E**) NAFLD activity score were quantified. Data are expressed as the mean with SEM for 8–10 mice per group. ## *p* < 0.001 compared to CDNW; ** *p* < 0.001 or * *p* < 0.05 compared to WDSW, vehicle control.

Histological analysis was carried out for all the groups. After 24 weeks, CDNW mice did not develop fatty liver, while WDSW mice with the vehicle control developed grade 3 macrovesicular steatosis. Increased lipid droplets (both in number and size) were very much evident, as evaluated by hematoxylin and eosin (H&E) staining in WDSW mice (Figure 4A), and *Tecomella undulata* treatment significantly reduced the number and size of the lipid droplets. Similar results were also confirmed by Oil Red O staining (Figure 4B). These WDSW mice showed significant steatosis (3 ± 0.1), hepatocellular ballooning (2 ± 0.2), and lobular inflammation (2.6 ± 0.25), with a mean with SEM NAFLD activity score (NAS) of 7.6 ± 0.3 confirming the development of NASH (Figure 5). 

Of note, *Tecomella undulata* treatment significantly reduced steatosis (1.6 ± 0.2), hepatocellular ballooning (1.4 ± 0.25), and lobular inflammation (1.8 ± 0.37). The NAFLD activity score in the *Tecomella undulata* treated mice was significantly lower (4.8 ± 0.6) compared to the WDSW group treated with the vehicle (Figure 5B,E). All of the histological scoring data were promising and were comparable to those of saroglitazar, the positive control group. 

### 2.5. Tecomella undulata Alleviates Hepatic ER Stress and Oxidative Stress

**Figure 6:**
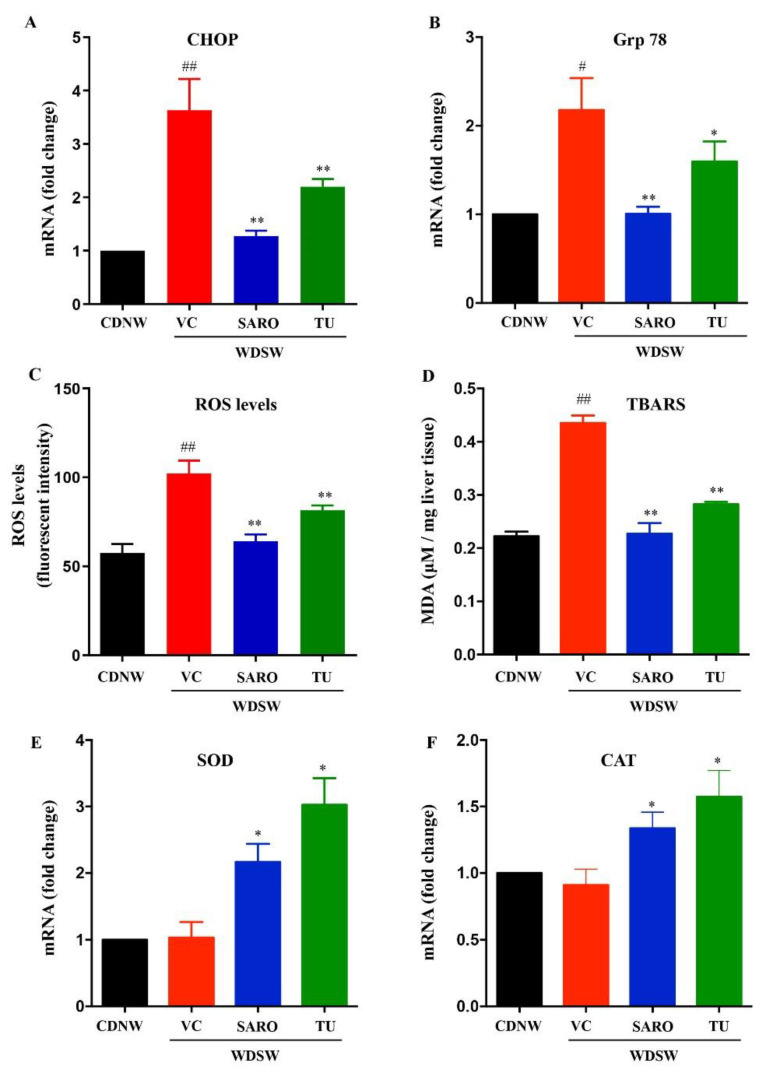
**Effect of *Tecomella undulata*** (**TU**) **on ER stress and oxidative stress in NASH mice.** The relative expression of hepatic mRNA levels of CHOP (**A**), Grp78 (**B**), SOD (**E**), and CAT (**F**) were determined using qRT-PCR. The experiments were carried out in triplicate and β-actin was used as an endogenous control for normalizing the mRNA levels. Intracellular ROS production (**C**) and lipid peroxidation (MDA levels) (**D**) were determined in the liver tissues of CDNW and WDSW mice treated with vehicle control (VC), saroglitazar (SARO) or *Tecomella undulata* (TU). Data are expressed as the mean with SEM for 8–10 mice per group. ## *p* < 0.001 or # *p* < 0.05 compared to CDNW; ** *p* < 0.001 or * *p* < 0.05 compared to WDSW, vehicle control. CHOP, C/EBP homologous protein; Grp78, 78 kDa glucose regulated protein; SOD, superoxide dismutase; CAT, catalase; ROS, reactive oxygen species; MDA, malondialdehyde.

The pathogenesis of NASH is characterized by chronic impairment of lipid metabolism, which results in cellular lipotoxicity, endoplasmic reticulum (ER) stress, lipid peroxidation, and mitochondrial dysfunction [24]. We examined whether administration of *Tecomella undulata* via oral gavage could prevent the progression of oxidative stress and ER stress, leading to improved liver function and reduced inflammation. WDSW mice showed a significant increase in ER stress, as measured by the expression of markers such as C/EBP homologous protein (CHOP) and 78-kDa glucose regulated protein (Grp78). However, *Tecomella undulata* significantly reduced the expression level of ER stress markers (Figure 6A,B). Furthermore, oxidative stress was determined by measuring the levels of reactive oxygen species (ROS) and malondialdehyde (MDA, a product of lipid peroxidation) in the liver tissues of the experimental mice. As expected, *Tecomella undulata* treated mice showed reduced levels of ROS and MDA in comparison to the WDSW mice treated with vehicle control (Figure 6C,D). Consistently, we observed increased levels of cellular antioxidants, as reflected by superoxide dismutase (SOD) and catalase (CAT) expression (Figure 6E,F) in *Tecomella undulata* treated WDSW mice compared to the vehicle control. 

### 2.6. Anti-Inflammatory Effects of Tecomella undulata in NASH

**Figure 7:**
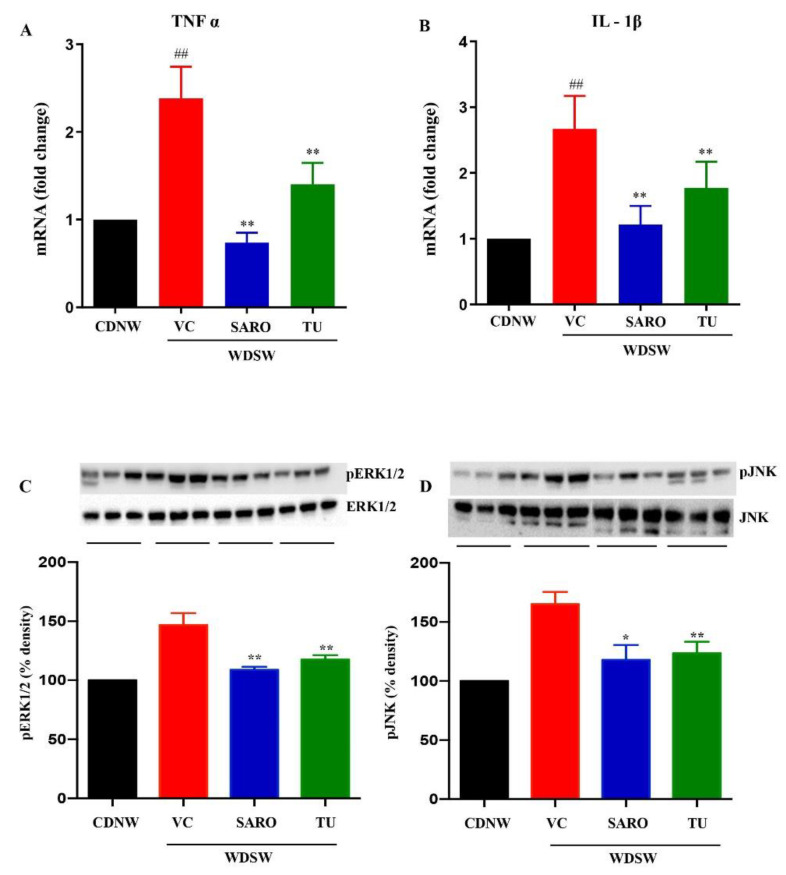
***Tecomella undulata*** (**TU**) **treatment ameliorated hepatic inflammation in NASH mice.** The relative expression of hepatic mRNA levels of TNFα (**A**), and IL-1β (**B**) were determined using qRT-PCR. The experiments were carried out in triplicate and β-actin was used as an endogenous control for normalizing the mRNA levels. Whole cell lysates were prepared from liver tissue of CDNW and WDSW treated with vehicle control (VC), saroglitazar (SARO) or *Tecomella undulata* (TU) for 12 weeks. Immunoblot analyses were performed for p-Erk1/2 and Erk1/2 (**C**), and p-JNK and JNK (**D**). Bar graphs show the densitometric values calculated after normalization to total Erk1/2 or total JNK. Data are expressed as the mean with SEM. ## *p* < 0.001 compared to CDNW; ** *p* < 0.001, * *p* < 0.05 compared to WDSW, vehicle control. TNFα, tumor necrosis factor α; IL-1β, interleukin- 1β; p-Erk1/2, phospho-extracellular signal-regulated protein kinase; p-JNK, phosphor- c-Jun N-terminal kinase.

Since inflammation plays a key role in the pathogenesis and progression of NASH, modulation of antioxidant responses may serve as a target to prevent the disease [25]. In this study, we investigated proinflammatory markers, tumor necrosis factor α (TNFα) and interleukin-1β (IL-1β) and found that *Tecomella undulata* administration to WDSW mice reduced inflammation (Figure 7B). Consistently, activated and phosphorylated c-Jun N-terminal kinase (JNK) and extracellular signal-regulated protein kinase (ERK1/2), which are primary stress kinases that play a fundamental role in the development of steatosis and are important players in inducing inflammation [26,27], were also found to be downregulated upon *Tecomella undulata* treatment (Figure 7C,D). All of these data were comparable to saroglitazar. 

**Figure 8:**
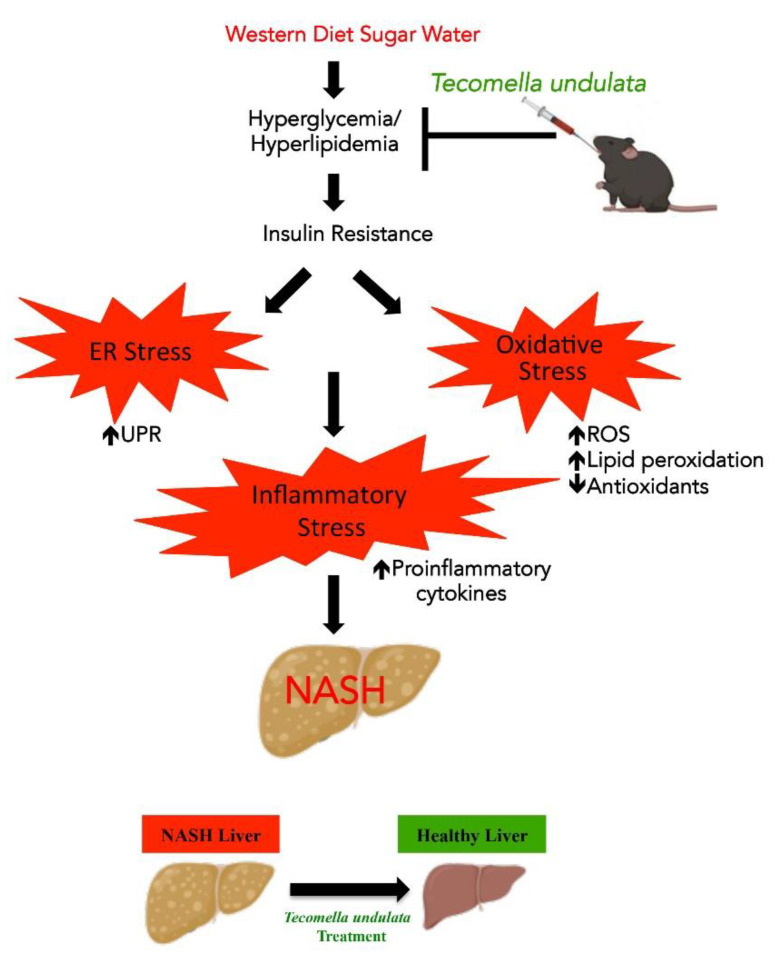
**Schematic representation depicting the mechanisms involved in *Tecomella undulata*** (**TU**)**-mediated amelioration of NASH.** Mice fed on WDSW for 12 weeks were treated with *Tecomella undulata* via oral gavage for another 12 weeks. Administration of *Tecomella undulata* ameliorated steatohepatitis in WDSW mice by improving insulin sensitivity, ER stress, oxidative stress, and increased production of antioxidants providing evidence that *Tecomella undulata* may have the potential to be used in the treatment of NASH.

Taken together, our results showed that *Tecomella undulata* could alleviate western diet sugar water-induced ER stress and oxidative stress, improve liver injury and antioxidant status, and thereby reduce inflammation (Figure 8).

## 3. Discussion

Steatohepatitis, a progressive form of NAFLD, is characterized by severe steatosis, inflammation, and cellular stress. The spectrum further continues to different stages of fibrosis and end-stage consequences, such as cirrhosis or hepatocellular carcinoma [28]. The primary treatment regimen focuses on NASH resolution, which includes several drug candidates, as well as regular physical activity and caloric restriction [29]. Due to NASH’s complexity and multifaceted pathological nature, there is an unmet need for the continued identification of novel therapeutics [30]. Hence, the use of herbal medications may therefore be a promising alternative with the potential for effective therapeutic benefit. To date, a number of plant extracts, polyherbal formulations, and phytochemicals have been examined for their potential therapeutic value in the treatment of various liver diseases, including NASH [31]. The current study provides proof of concept that *Tecomella undulata* improves histological characteristics, as well as the key biological processes that are associated with the pathogenesis of NASH. 

Despite the fact that there is literature on the preclinical evaluation of herbal remedies for the treatment of NASH, the primary flaw in many studies lies in not making the right choice of the animal model. The quality of the diet, animal strain, concentration of fat, cholesterol, and sugar, time period, and reproducibility are the key characteristics that are always overlooked in NAFLD research. There are a wide variety of genetic and dietary animal models that are available to study NASH [22]. However, choosing the ideal animal model mimicking human NASH with respect to diet-induced obesity and insulin resistance is crucial, without much alteration in the macronutrient composition (deficiency or overnutrition) or the addition of toxins. In this study, we have used a mouse model (detailed in Section 4) that not only recapitulates the effects of the diet but also demonstrates concordance with histology, disease progression, biological processes, and pathways. With the exception of the fact that steatohepatitis takes around 24 weeks to manifest, the mice exhibit significant translatability to human NASH, as they progressively develop obesity, insulin resistance, dyslipidemia, and inflammation. In addition to the experimental NASH model, other crucial elements, including dosage, timing of the intervention, route of administration, duration of exposure, and endpoint assessments, were carefully chosen to assess the efficacy of *Tecomella undulata* for the treatment of NASH. 

In this study, we provide in vivo evidence for the hepatoprotective effects of *Tecomella undulata* in ameliorating NASH. *Tecomella undulata* is known to have a special place in herbal medicine and has been widely recommended to treat hepatosplenomegaly, hepatitis, and obesity [16,32]. Studies have investigated the pharmacological effects of *Tecomella undulata* and have reported hepatoprotective, anti-inflammatory, immunomodulatory, and antimicrobial activities [33,34,35]. However, there is a lack of scientific data illuminating the molecular processes through which *Tecomella undulata* exerts its effects in preclinical research using an appropriate experimental model. To our knowledge, this is the first study to investigate the effect of *Tecomella undulata* on hepatic damage caused by western diet sugar water. We evaluated the protective effects of *Tecomella undulata* against hepatic lipid accumulation in an experimental NASH model and how it regulates the genes and proteins involved in biological processes associated with the development and progression of NASH. The C57bl/6 mice developed fatty liver after 12 weeks, following the initiation of a western diet sugar water regimen, and progressed to NASH, in concordant with human NASH. The administration of *Tecomella undulata* to the mice resulted in improvement in obesity, as marked by a reduction in body weight and liver weight. There was also a significant amelioration in insulin resistance. Furthermore, we also demonstrated a marked improvement in the histological and biochemical outcomes after treatment with *Tecomella undulata* in NASH mice. Collectively, it was interesting to note that the hepatoprotective effects of *Tecomella undulata* followed a similar trend to those of saroglitazar, a dual peroxisome proliferator activated receptor α/γ agonist approved for the treatment of human NASH [36,37]. 

Previous studies have shown the hepatoprotective activity of ethanolic and methanolic extracts of *Tecomella undulata* in rats against thioacetamide- and carbon tetrachloride-induced hepatocyte damage, respectively [32,38]. Ethanolic extracts of *Tecomella undulata* have shown anti-hyperglycemic and antioxidant potential in streptozotocin–nicotinamide-induced type 2 diabetic rats, although the mechanism remains ill defined [39]. In vitro and in vivo studies of *Tecomella undulata* ethyl acetate extract revealed anti-obesity efficacy by downregulating adipogenesis and lipogenesis via sirtuin 1 (SIRT1), adiponectin, and peroxisome proliferator activated receptor γ (PPARγ) [16]. However, the current study provides insight into the mechanisms by which *Tecomella undulata* may mitigate NASH. Hepatocellular stress plays a prominent role in the onset of NASH. The large influx of free fatty acids saturates the oxidation mechanisms, leading to the production of reactive oxygen species (ROS) which are not neutralized by antioxidants, resulting in hepatic injury via the Ras/Raf/Erk pathway [27,40]. Toxic lipid species, such as palmitic acid, accumulate in the liver and cause ER stress, as evidenced by the unfolded protein response (UPR) [41,42]. Thus, markers for ER stress (Grp78 and CHOP) and oxidative stress (ROS and MDA levels) were studied, along with ERK1/2, and we found that treatment with *Tecomella undulata* reduced oxidative stress and ER stress in NASH mice, providing a rationale for low cholesterol and lipid reduction in the liver. As expected, antioxidant levels were increased due to the treatment, as shown by SOD1 and CAT expression. The hepatic inflammatory response is a major driver of NAFL progression to NASH and fibrosis. Several intra- and extrahepatic factors trigger the inflammatory system, which exacerbates cellular injury and the apoptotic response. Along with inflammatory mediators such as TNF-α and IL-1β, free cholesterol and oxidative stress can activate JNK-dependent proinflammatory pathways [43,44,45]. Our study reports the anti-inflammatory potential of *Tecomella undulata,* as indicated by the marked reduction in cellular mediators of inflammation.

This study adds to the growing literature on the use of herbal medicines and supports the further assessment of *Tecomella undulata* in humans for the treatment of NASH. Previous studies have used analytical HPLC, which has led to the identification of betulinic acid, ferulic acid, rutin, quercetin, vanillic acid, and another nine chemicals in methanol or ethanol extract of *Tecomella undulata’s* stem bark [16,32,46], but unfortunately no validation has been performed in NASH. The present study provides the foundations by presenting strong evidence for the traditional use of *Tecomella undulata* in herbal medicine with well designed reverse pharmacology-guided research proving its safety and therapeutic efficacy. Based on previous HPLC studies, future experiments are in planned to isolate and purify potent hit compounds and also perform therapeutic validation of these pure compounds in the treatment of NASH, rather than examining only the extract from the stem bark of *Tecomella undulata*. We also plan to conduct large-scale randomized controlled trials (RCT) to support the therapeutic effects of Tecomella undulata in NASH. We strongly believe that the application of omics analysis (transcriptomics, proteomics, lipidomics, metabolomics, and metagenomics) may also aid in exploring the molecular mechanisms in our studies. 

## 4. Materials and Methods

### 4.1. Chemicals

The saroglitazar was a generous gift from the Zydus Research Center, Cadila Healthcare Limited, Ahmedabad, Gujarat, India. Antibodies to JNK and ERK1/2 were procured from Santa Cruz Biotechnology, Inc. (Santa Cruz, CA, USA); the mouse insulin ELISA kit from Krishgen Biosystems; the ROS Detection Assay Kit from BioVision (Waltham, MA, USA); and the TBARS assay kit from Cayman Chemicals (Ann Arbor, MI, USA). The western diet was customized and purchased from Research Diets Inc. (New Brunswick, NJ, USA), and glucose and fructose were procured from Himedia Laboratories (Mumbai, India).

### 4.2. Plant Material

*Tecomella undulata* crude powder was purchased from Ayur Drugs & Pharmaceuticals, Mysore, India. As per the details provided by the company, the bark of *Tecomella Undulata* was collected from the natural habitat of Jaipur, Rajasthan. We confirmed the authentication with the JSS Ayurveda Medical College and Hospital and Government Ayurveda Medical College, Mysuru. The plant material was also authenticated by comparison with the reference specimen (EP 427), preserved at the herbarium of the National Bureau of Plant Genetic Resources. Furthermore, the stem bark powder of *Tecomella Undulata* was subjected to pharmacopoeia tests.

### 4.3. Cell Culture

HepG2 and Huh7 cells were cultured in DMEM (Himedia Laboratories, Mumbai, India) containing 1 g/L and 4.5 g/L glucose, respectively, supplemented with 10% fetal bovine serum (Gibco, USA), L-glutamine, sodium pyruvate, nonessential amino acids, and 100 U/mL penicillin–streptomycin incubated at 37 °C in 5% CO_2_.

### 4.4. In Vitro Cytotoxicity Assay

A WST-1 assay (Takara, Shiga, Japan) was performed on HepG2 cells and Huh7 cells to assess the cellular cytotoxicity of *Tecomella undulata*. Briefly, 1 × 10^4^ cells were seeded into a 96-well plate and cultured up to 70% confluency. Cells were treated with *Tecomella undulata* powder dissolved in methanol at different concentrations (0, 10, 20, 50, 100, 250, 500, 750, 1000 μg/mL) for 24 h, 48 h, and 72 h. At the end of the treatment, WST-1 assay mix (1:10 dilution) was added to each well and incubated for 1 h at 37 °C in 5% CO_2_. The absorbance was measured at 450 nm on a multi-mode plate reader (EnSpire™ Multimode Plate Reader, Perkin Elmer, Waltham, MA, USA). Percent cell viability was calculated in comparison to the control (no treatment).

### 4.5. Animal Model

The 6–7 week-old male C57Bl/6 mice (Charles River Laboratories, Wilmington, MA, USA) used in the study (total of 60 mice) were maintained as described previously [20,47]. All mice were housed on a 12 h light-dark cycle at 21–24 °C in an animal facility administered by the Centre for Experimental Pharmacology and Toxicology, JSS AHER. The mice were regularly monitored for body weight, food and water intake, and were killed at varied time points following dietary and drug interventions. Blood collection and tissue harvesting were performed according to the approved protocols, in accordance with the guidelines and regulations of the Committee for the Purpose of Control and Supervision of Experiments on Animals (CPCSEA), Government of India (JSSAHER/CPT/IAEC/060/2021).

### 4.6. Study Design and Interventions

After a week of acclimatization, the mice were fed with either a chow diet (CD) or a western diet (WD) with high fat, high sucrose, and high cholesterol (WD, 21% fat, 41% sucrose, and 1.25% cholesterol by weight, Research Diet, Inc. USA) along with normal water (NW) or high sugar (SW, 18.9 g/L d-glucose and 23.1 g/L d-fructose) containing drinking water ad libitum for 12 weeks. *Tecomella undulata* and saroglitazar were dissolved in 0.5% carboxymethylcellulose (CMC) and Tween 80 in a 99.5:0.5 ratio and administered via oral gavage once a day for 12 weeks. Saroglitazar was used as a positive control throughout the study. After 12 weeks, mice were randomly divided into 6 groups (Supplementary Figure S1). There were the following three groups of chow diet fed mice: CDNW with vehicle control-VC (0.5% CMC and Tween 80 in a 99.5:0.5 ratio), CDNW with saroglitazar (4 mg/kg/day), and CDNW with *Tecomella undulata* (80 mg/kg/day). There were the following three groups of western diet-fed mice: WDSW with vehicle control-VC (0.5% CMC and Tween 80 in a 99.5:0.5 ratio), WDSW with saroglitazar (4 mg/kg/day), and WDSW with *Tecomella undulata* (80 mg/kg/day). The mice were constantly monitored for food and water intake, and their body weight was recorded on a weekly basis. At the end of treatment (24 weeks), mice were killed, blood was collected and biochemical analysis was performed. 

### 4.7. Choice of Tecomella undulata Dosage

Pharmacopoeia tests were performed to ensure the pharmaceutical quality of *Tecomella undulata* stem bark powder for use in in vivo research (Supplementary Table S1). The dose of *Tecomella undulata* was based on the body surface area (BSA) conversion factor as recommended by the Food and Drug Administration of the US federal government [48]. A dose of 80 mg of *Tecomella undulata*/kg body weight of mice was calculated based on the equation: therapeutic human dose of *Tecomella undulata* (6 g/kg) × body surface area (BSA) conversion factor for mice (0.0026) × inter species conversion factor (5). 

### 4.8. Blood Collection and Biochemical Analysis

Bood was collected via retro-orbital puncture from overnight fasted mice prior to killing. It was then allowed to clot at room temperature and centrifuged at 1500× *g* for 15 min at 4 °C. The serum was collected and stored at −80 °C until further analysis. Serum AST, ALT, cholesterol, triglycerides, and LDL-cholesterol were measured using kits from Monlab Tests, Barcelona, Spain, according to the manufacturer’s instructions.

### 4.9. Glucose Tolerance Test (GTT) and Insulin Tolerance Test (ITT)

A glucose tolerance test was performed on overnight fasted mice by measuring the baseline blood glucose level with an Accu-chek Glucometer (Roche Diagnostics, Rotkreuz, Switzerland). Glucose (1 g/kg body weight) dissolved in phosphate buffered saline was intraperitoneally injected into mice. After a glucose injection, blood glucose levels were measured at various time intervals (0, 15, 30, 60, 90, and 120 min). An insulin tolerance test was performed by fasting the mice for 5–6 h and measuring the baseline glucose levels, in a similar way to GTT. Mice were intraperitoneally injected with insulin (0.75 U/kg body weight). Blood glucose was measured thereafter at different time intervals (0, 15, 30, 45, 60, 90, and 120 min). GTT and ITT were performed before starting the compound treatment (12 weeks) and also at the end of the treatment (24 weeks). 

### 4.10. Histological Analysis

Paraffin-embedded formalin-fixed liver tissues were subjected to hematoxylin and eosin (H&E) staining to assess steatosis, hepatocyte ballooning, and lobular inflammation. A pathologist at JSS Hospital scored the liver specimens (blindfolded) for severity of NAFLD (following the FLIP algorithm and NASH Clinical Research Network (CRN) criteria [49,50]. Oil Red O staining was performed on fresh liver sections to evaluate steatosis, using established protocols.

### 4.11. TBARS Assay

Lipid peroxidation was estimated by the TBARS assay, following the manufacturer’s instructions. The production of TBARS was expressed as the equivalent of malondialdehyde (MDA). Liver tissue (25 mg) liver tissue was weighed and homogenized. Tissue homogenate was used to measure the MDA levels. The reaction products were measured at 530 nm excitation and 550 nm emission. The MDA levels were normalized against standard values.

### 4.12. ROS Assay

Intracellular reactive oxygen species production was determined in liver tissues by measuring 2’, 7’-dichlorofluorescein diacetate (DCFH-DA) (BioVision, Milpitas, CA, USA), following the manufacturer’s instructions. A single cell suspension was prepared from fresh liver tissue by enzyme digestion and pelleted cells were incubated with ROX label for 45 min at 37 °C in the dark. After incubation, fluorescence intensity was measured at excitation/emission = 495/529 nm using a multi-mode plate reader (EnSpire™ Multimode Plate Reader, Perkin Elmer, Waltham, MA, USA) and the change in florescence was determined after background subtraction. 

### 4.13. RNA Isolation and Quantitative Real Time PCR

Total RNA was isolated from frozen liver tissue using TRIzol (Sigma, San Jose, CA, USA). A NanoDrop spectrophotometer was used to determine the concentration and quality of RNA. The RNA was reverse transcribed into cDNA using a Verso cDNA synthesis kit (Thermo, Waltham, MA, USA), following the manufacturer’s instructions. A Rotor-Gene Q 5plex HRM system (QIAGEN, Hilden, Germany) was used to perform quantitative real-time PCR. The reactions were carried out using a DyNamo Colorflash SYBR green kit (Thermo, Waltham, MA, USA), 0.5 mM primers (IDT), and 50 ng of cDNA in a 20 μL reaction volume. The relative fold change in mRNA levels was calculated as 2^−ΔΔCt^ and was expressed normalized with endogenous control β-actin. 

### 4.14. Western Blot

Liver tissue lysates were prepared by solubilizing the tissue in RIPA buffer (Sigma, USA) containing protease/phosphatase inhibitor (Thermo Fisher, Waltham, MA, USA). Homogenized tissues were centrifuged at 13,000 rpm for 10 min at 4 °C and the supernatants were collected. The protein concentration of the tissue lysates was estimated with Bradford’s protein estimation method, using Bio Rad Protein Assay Dye Reagent (Bio Rad, Hercules, CA, USA). To separate the proteins, 30 μg of protein samples were loaded into SDS-PAGE and transferred onto a nitrocellulose membrane for all western blots. The membranes were further blocked using 5% nonfat skim milk for 1 h and probed with specific primary antibodies (pErk1/2, Erk1/2, pJNK, JNK, Santa Cruz) and β-actin (Cell Signaling). After overnight incubation at 4 °C, membranes were incubated with secondary antibodies (Cell Signaling) for 2 h at room temperature. The blots were visualized using a Western Bright ECL HRP substrate (Advansta, San Jose, CA, USA). The blot images were analyzed using ImageJ software 1.53t. The intensity of each band was normalized with endogenous control, β-actin.

### 4.15. Statistical Analysis

Results were calculated as mean with SEM. Statistical significance was analyzed using Student’s *t*-test for two groups or analysis of variance (ANOVA) with post hoc Bonferroni correction for multiple comparisons. All statistical analyses were performed using GraphPad Prism software (version 6), and *p* values < 0.05 (*) or < 0.001 (**) were considered significant. In all cases, *n* = 8–10 mice per group, unless otherwise indicated in the figure legends. 

## 5. Conclusions

In summary, we first discovered the promising hepatoprotective effects of the stem bark powder of *Tecomella undulata* in a preclinical model of NASH that closely mimics the dietary pattern, systemic milieu, and histological spectrum of NASH, and also shows activation of the key cellular pathways linked to human disease. Our study demonstrated that *Tecomella undulata* improves insulin sensitivity by attenuating oxidative stress, ER stress, and inflammation in steatohepatitis. Thus, *Tecomella undulata* could have a therapeutic role in the prevention of NASH. 

## Data Availability

Not applicable.

## Supplementary Materials

The following supporting information can be downloaded at: https://www.mdpi.com/article/10.3390/ijms24043244/s1.

## Abbreviations

NAFLD, nonalcoholic fatty liver disease; NASH, nonalcoholic steatohepatitis; ROS, reactive oxygen species; ER, endoplasmic reticulum; CD, chow diet; WD, western diet; NW, normal water; SW, sugar water; CMC, carboxymethylcellulose; AST, aspartate transaminase; ALT, alanine transaminase; LDL, low density lipoprotein; GTT, glucose tolerance test; ITT, insulin tolerance test; TBARS, thiobarbituric acid reactive substances; MDA, malondialdehyde; HOMA-IR, homeostatic model assessment for insulin resistance; NAS, NAFLD activity score; CHOP, C/EBP homologous protein; Grp78, 78-kDa glucose regulated protein; SOD, superoxide dismutase; CAT, catalase; TNFα, tumor necrosis factor α; IL-1β, interleukin-1β; JNK, c-Jun N-terminal kinase; ERK1/2, extracellular signal-regulated protein kinase; SIRT1, sirtuin 1; UPR, unfolded protein response; PPAR γ, peroxisome proliferator activated receptor γ; VC, vehicle control; SARO, saroglitazar; TU, *Tecomella undulata*; SEM, standard error of the mean.
