# Supplementary material for: Unraveling the Potential Role of Tecomella undulata in Experimental NASH
Source: Int J Mol Sci. 2023 Feb 7;24(4):3244. doi: 10.3390/ijms24043244 (PMC9962064; doi:10.3390/ijms24043244)
Supplement: Supplementary file 1 [file ijms-24-03244-s001.zip › Supplementary Figure Legends.pdf]

Supplementary Figure S1. Study design to evaluate the efficacy of *Tecomella undulata* (TU) in the treatment of NASH.

Supplementary Figure S2. Cell viability after treatment with *Tecomella undulata* (TU) in Huh7 and HepG2 cells. WTS-1 assay were performed to assess the cellular cytotoxicity of *Tecomella undulata*. Various concentrations of *Tecomella undulata* (10-1000 μM) were treated to HepG2 and Huh7 cells for 24 hours. Based on the cell viability, cells were further treated with 50μM or 100 μM for 24, 48 and 72 hours. Percent cell viability was calculated in comparison to the control (no treatment).

Supplementary Figure S3. Representative photographs (**A**) and gross anatomy of liver (**B**) from mice fed with CDNW and WDSW treated with or without *Tecomella undulata* (TU), showing discrepancy in body size, and liver morphology and size. Vehicle control (VC), Saroglitazar (SARO).

Supplementary Figure S4. Effect of *Tecomella undulata* (TU) on bodyweight, liver weight, and insulin sensitivity on CDNW mice. Mice were treated with CDNW with vehicle control (VC), Saroglitazar (SARO) or *Tecomella undulata* (TU) via oral gavage for 24 weeks. At the completion of the treatment, body weights (**A**), and liver weight (**B**) were measured. Mice were fasted overnight and blood glucose concentration (mg/dl) was measured after intraperitoneal injection of 1g/kg glucose (**C**). Mice fasted for 4-5 hours were administered 0.75 units/kg insulin and blood glucose concentration (mg/dl) measured (**D**). The bar graphs depict the area under curve (AUC) with or without treatment. Data are expressed as mean with SEM for 8-10 mice per group.

Supplementary Figure S5. *Tecomella undulata* (TU) treatment has no effect on fasting glucose and insulin. Mice were treated with CDNW with vehicle control (VC), Saroglitazar (SARO) or *Tecomella undulata* (TU) via oral gavage for 24 weeks. At the completion of the treatment, fasting glucose (**A**), and fasting insulin (**B**) were measured. HOMA-IR (**C**) was calculated using the formula: (fasting insulin (milliunits/liter)×fasting glucose (mmol/liter))/22.5. Data are expressed as mean with SEM for 8-10 mice per group.

Supplementary Figure S6. Effect of *Tecomella undulata* (TU) treatment on liver enzymes and lipid profile in mice fed with CDNW. Serum biochemical analyses were performed on CDNW mice treated with vehicle control (VC), Saroglitazar (SARO) or *Tecomella undulata* (TU) for 24 weeks. Mice were fasted overnight and blood was collected. (**A**) ALT, (**B**) ALT, (**C**) triglycerides, (**D**) cholesterol, and (**E**) serum LDL-C. Data are expressed as the mean with SEM for 8–10 mice per group. AST, aspartate aminotransferase; ALT, alanine aminotransferase; LDL-C, low-density lipoprotein-Cholesterol.

Supplementary Table S1. Results of Pharmacopeia analysis on *Tecomella undulata* (TU) stem bark powder sample.
