# Supplementary material for: Unraveling the Potential Role of Tecomella undulata in Experimental NASH
Source: Int J Mol Sci. 2023 Feb 7;24(4):3244. doi: 10.3390/ijms24043244 (PMC9962064; doi:10.3390/ijms24043244)
Supplement: Supplementary file 1 [file ijms-24-03244-s001.zip › Supplementary Table S1.pdf]

Supplementary Table S1: Results of Pharmacopeia analysis

Sl. No.	Test	Unit	Result	Ayurvedic Pharmacopoeia of India	Test Method
	**Physiochemical**				
1	Color	-	Brown color powder	Brown color	GCAS/FC/SOP/I7.2-G
2	Odor	-	Characteristic odor	Indistinct odor	
3	Taste	-	Characteristic taste	Indistinct Taste	
4	Total Ash	%	6.64	12.0 max	IS 3077:1992
5	Acid insoluble Ash	%	0.34	1.0 max	
6	Water soluble extractive	%	19.27	10.0 max	
7	Alcohol soluble extractive	%	18.83	15.0 max	
	**Residue - Metals**				
1	Lead as Pb	mg/kg	BDL (DL: 0.01)	10 max	GCAS/FC/SOP-21-M
2	Cadmium as Cd	mg/kg	BDL (DL: 0.01)	0.3 max	
3	Mercury as Hg	mg/kg	BDL (DL: 0.01)	1.0 max	
4	Arsenic as As	mg/kg	BDL (DL: 0.01)	3.0 max	
	**Biological**				
1	Total Bacterial count	Cfu/g	$65x10^2$	$<10^5$	IS 5402:2012 RA 2018
2	Yeast and Mold count	Cfu/g	$20x10^1$	$<10^3$	IS 5403:1999 RA 2018
3	Enetrobacteriaceae	Cfu/g	<10	Not Specified	IS 17112 (P-2):2019
	**Toxins**				
1	Total Aflatoxins (B1, B2, G1, G2)	µg/ kg	BDL (DL: 2.5)	<5.0	FSSAI manual methods - Mycotoxin
	**Residue - Pesticides**				
1	Alachlor	mg/kg	BDL (DL: 0.01)	0.02 max	AOAC 18th Edition: 2005, 990.06 & 991.07
2	Atrazine	mg/kg	BDL (DL: 0.01)	Not Specified	
3	Aldrin/Dieldrin	mg/kg	BDL (DL: 0.01)	0.05 max	
4	Alpha HCH	mg/kg	BDL (DL: 0.01)	0.3 max	
5	Beta HCH	mg/kg	BDL (DL: 0.01)	0.3 max	
6	Butachlor	mg/kg	BDL (DL: 0.01)	Not Specified	
7	Chloropyriphos	mg/kg	BDL (DL: 0.01)	0.2 max	
8	Delta HCH	mg/kg	BDL (DL: 0.01)	0.3 max	
9	2,4-Diachlorophenoxyacetic acid	mg/kg	BDL (DL: 0.01)	Not Specified	
10	DDT (o, p and p, p – Isomers of DDT, DDE and DDD)	mg/kg	BDL (DL: 0.01)	1.0 max	
11	Endosulfan (Alpha, Beta and Sulphate)	mg/kg	BDL (DL: 0.01)	3.0 max	
12	Ethion	mg/kg	BDL (DL: 0.01)	2.0 max	
13	Gamma – HCH (Lindane)	mg/kg	BDL (DL: 0.01)	0.6 max	
14	Isoproturon	mg/kg	BDL (DL: 0.01)	Not Specified	
15	Malathion	mg/kg	BDL (DL: 0.01)	1.0 max	
16	Methyl parathion	mg/kg	BDL (DL: 0.01)	0.2 max	
17	Monocrotophos	mg/kg	BDL (DL: 0.01)	Not specified	
18	Phorate	mg/kg	BDL (DL: 0.01)	Not specified	

Note: BDL: Below Detection Limit. DL: Detection Limit.
