# Supplementary figures and images for: Unraveling the Potential Role of Tecomella undulata in Experimental NASH
Source: Int J Mol Sci. 2023 Feb 7;24(4):3244. doi: 10.3390/ijms24043244 (PMC9962064; doi:10.3390/ijms24043244)

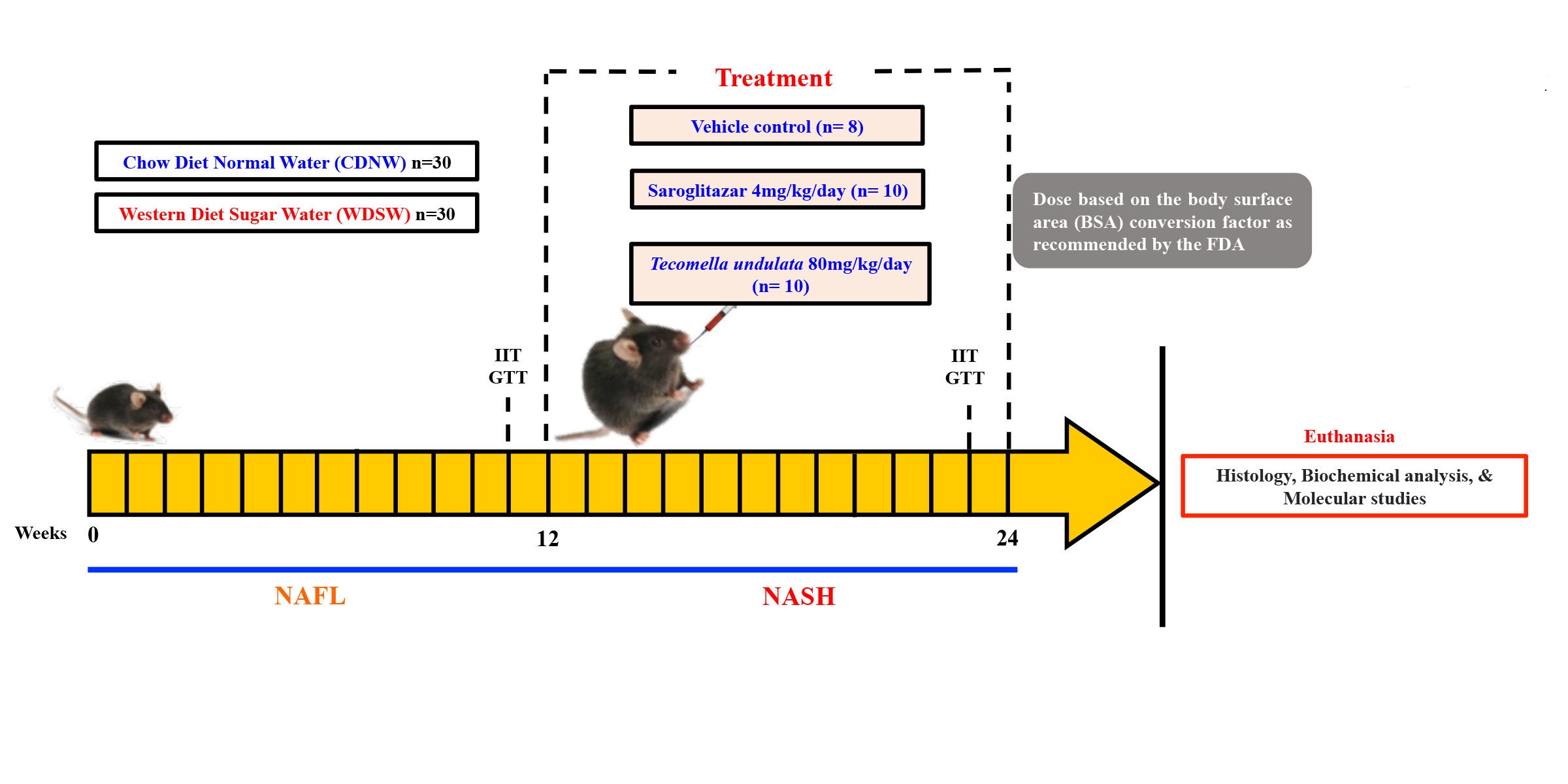

Supplement: Supplementary file 1 [file ijms-24-03244-s001.zip › Supplementary Figure S1.jpg]

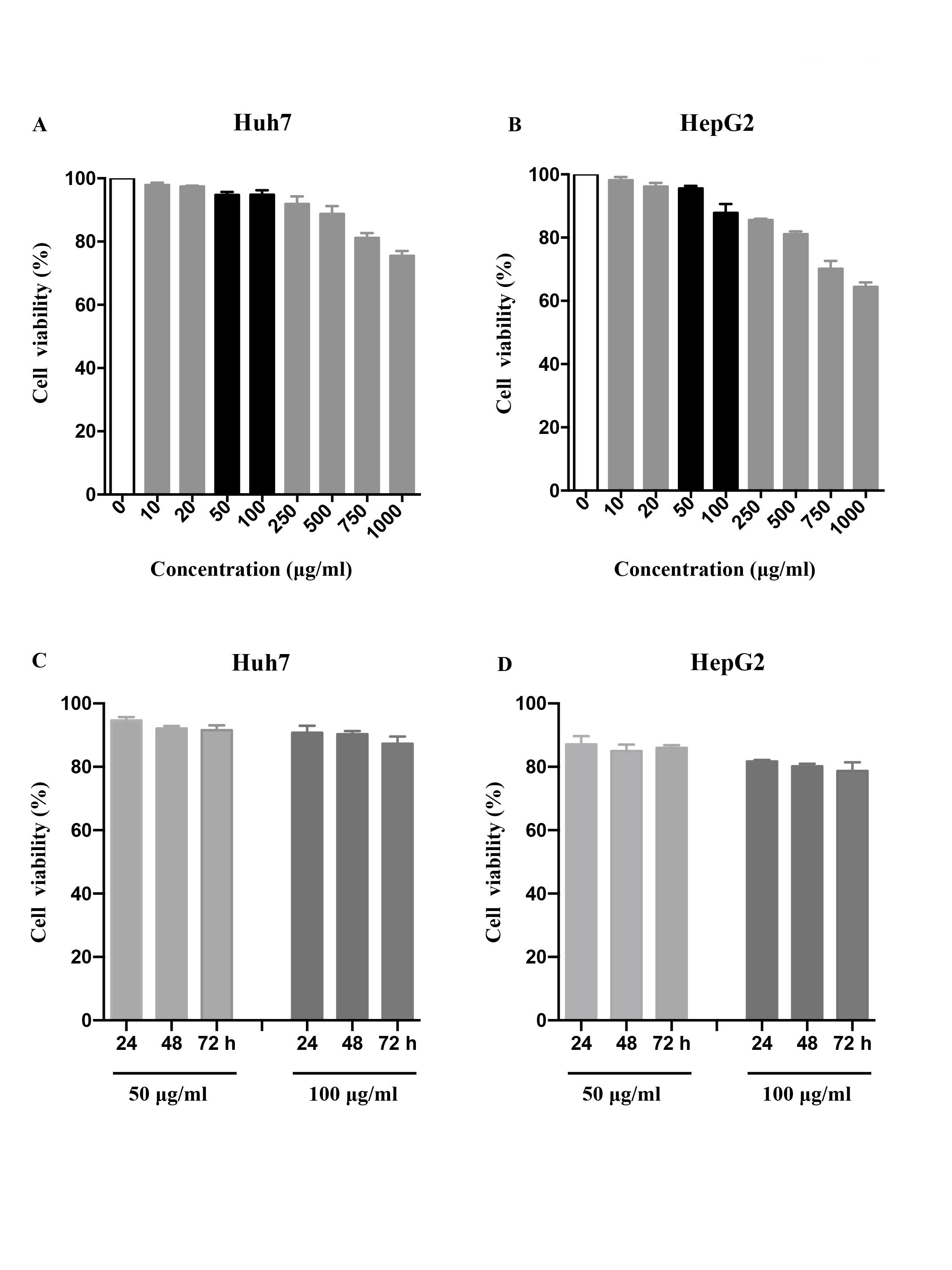

Supplement: Supplementary file 1 [file ijms-24-03244-s001.zip › Supplementary Figure S2.jpg]

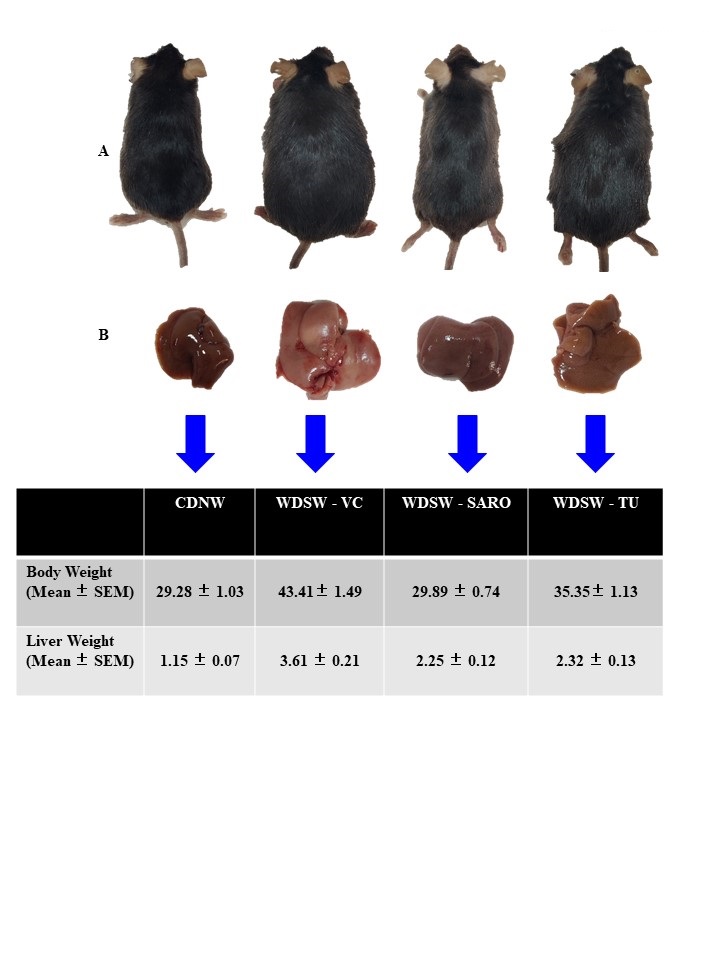

Supplement: Supplementary file 1 [file ijms-24-03244-s001.zip › Supplementary Figure S3.jpg]

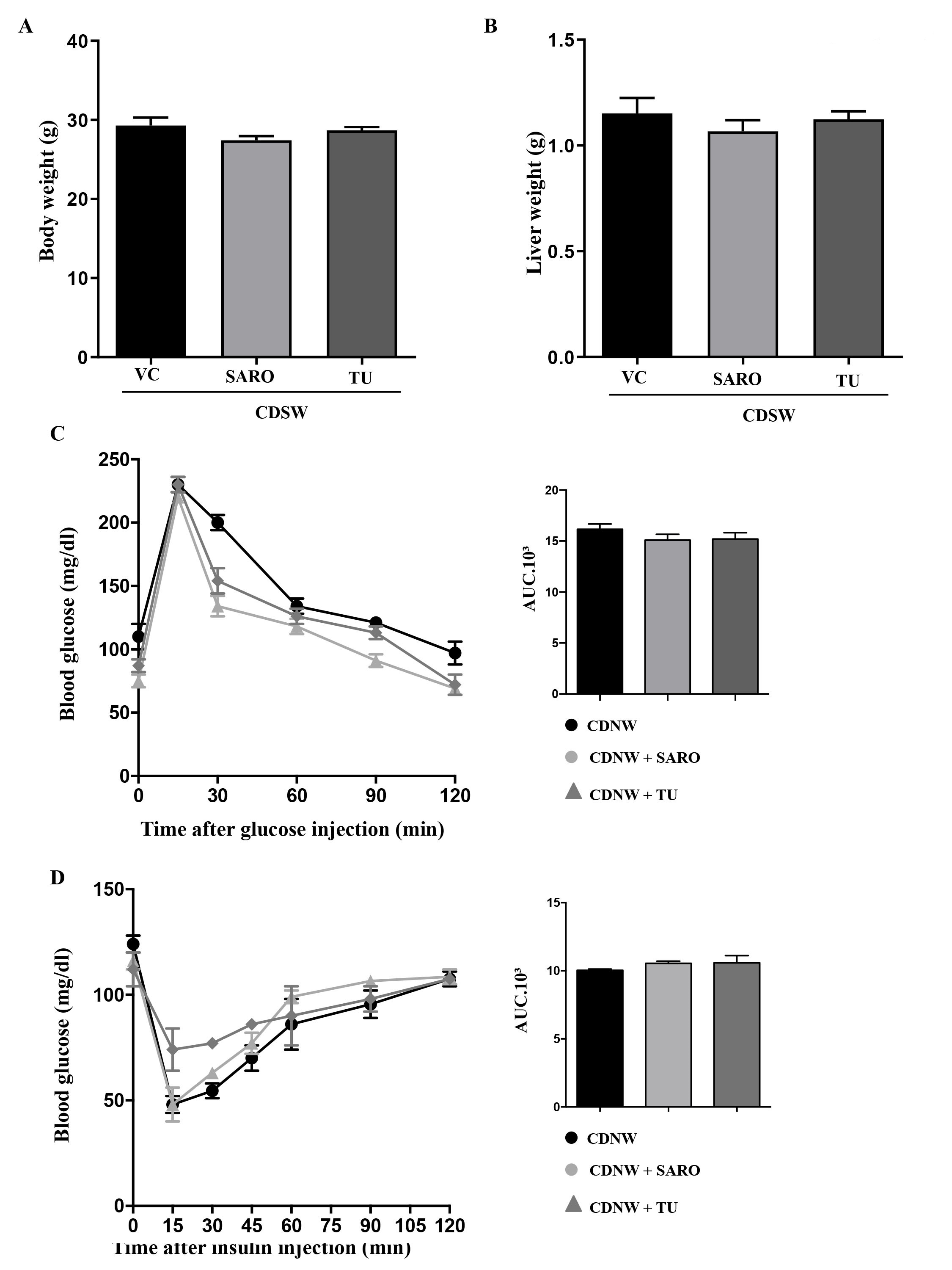

Supplement: Supplementary file 1 [file ijms-24-03244-s001.zip › Supplementary Figure S4.jpg]

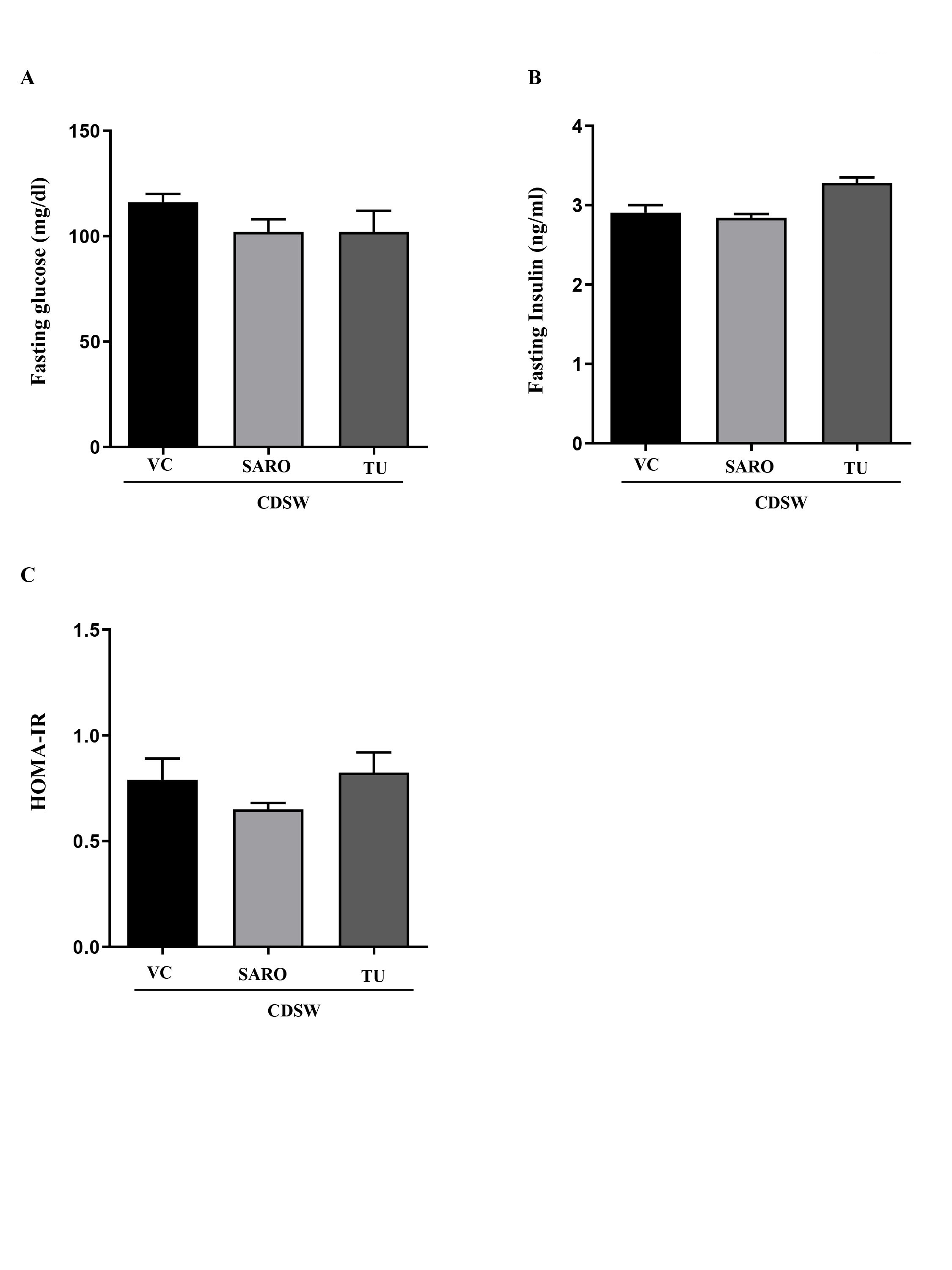

Supplement: Supplementary file 1 [file ijms-24-03244-s001.zip › Supplementary Figure S5.jpg]

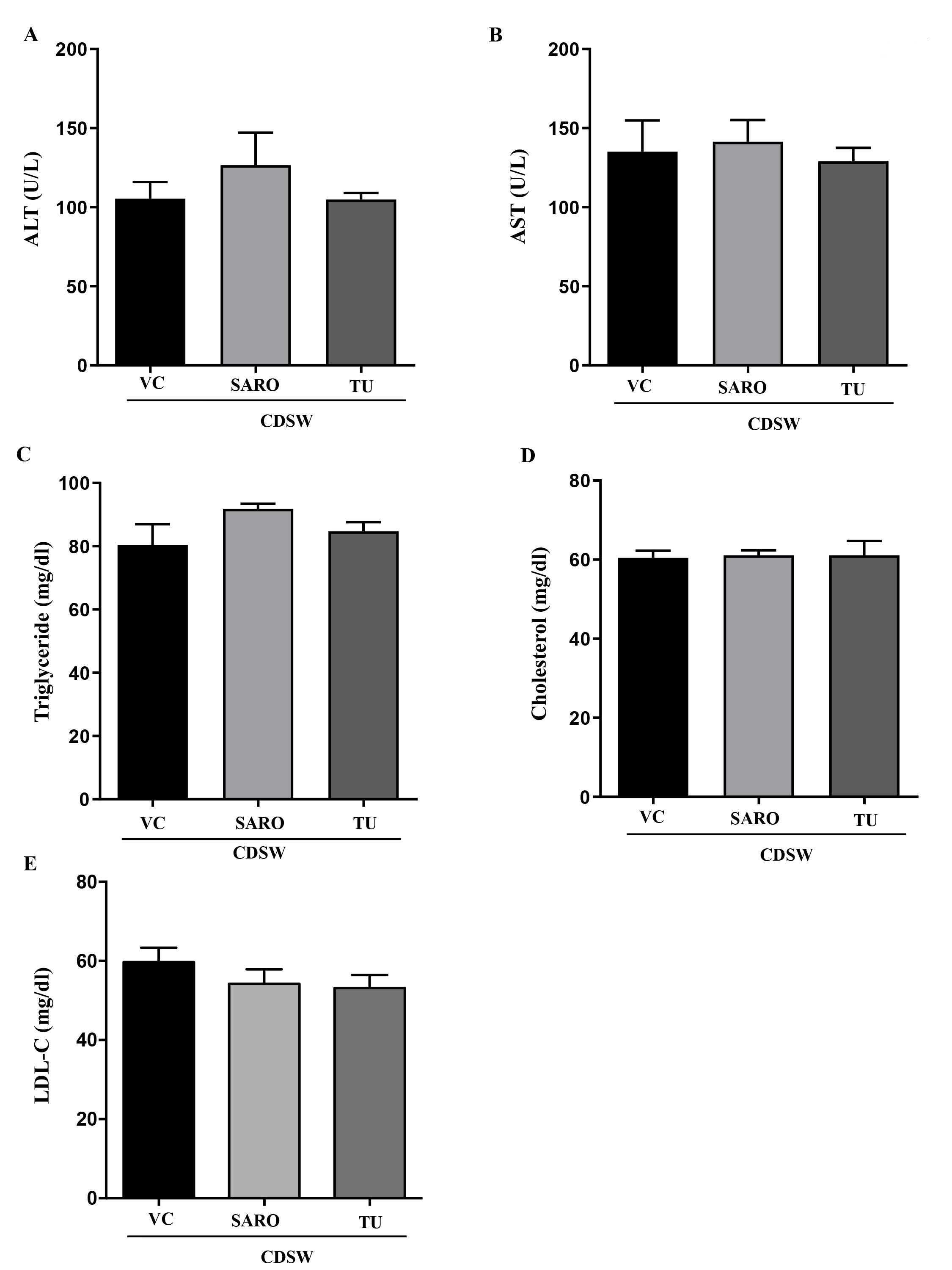

Supplement: Supplementary file 1 [file ijms-24-03244-s001.zip › Supplementary Figure S6.jpg]
